# Electronic Structure and Hole Transfer of All B-DNA Dimers and Homopolymers, via the Fishbone-Wire Model

**DOI:** 10.3390/ma16083200

**Published:** 2023-04-18

**Authors:** Constantinos Simserides, Aikaterini Orfanaki, Neokleia Margariti, Konstantinos Lambropoulos

**Affiliations:** Department of Physics, National and Kapodistrian University of Athens, GR-15784 Athens, Greece

**Keywords:** B-DNA, DNA, dimer, homopolymer, oxidation, hole, deoxyribose, base pair, transfer, Fishbone-Wire Model, tight binding

## Abstract

We employ the Tight Binding Fishbone-Wire Model to study the electronic structure and coherent transfer of a hole (the absence of an electron created by oxidation) in all possible ideal B-DNA dimers as well as in homopolymers (one base pair repeated along the whole sequence with purine on purine). The sites considered are the base pairs and the deoxyriboses, with no backbone disorder. For the time-independent problem, we calculate the eigenspectra and the density of states. For the time-dependent problem after oxidation (i.e., the creation of a hole either at a base pair or at a deoxyribose), we calculate the mean-over-time probabilities to find the hole at each site and establish the frequency content of coherent carrier transfer by computing the Weighted Mean Frequency at each site and the Total Weighted Mean Frequency of a dimer or polymer. We also evaluate the main oscillation frequencies of the dipole moment along the macromolecule axis and the relevant amplitudes. Finally, we focus on the mean transfer rates from an initial site to all others. We study the dependence of these quantities on the number of monomers that are used to construct the polymer. Since the value of the interaction integral between base pairs and deoxyriboses is not well-established, we treat it as a variable and examine its influence on the calculated quantities.

## 1. Introduction

The purpose of this work is to establish the main features of the Fishbone-Wire Tight Binding (TB) model for B-DNA dimers and homopolymers. The Fishbone-Wire Model (FWM) and the Fishbone-Ladder Model for DNA were introduced a few years ago now. In Ref. [1], the authors modeled environmental fluctuations by varying the on-site energies of the backbone sites under some simplifying assumptions regarding the on-site energies of the bases or base pairs and the interaction integrals between them. Similar models have been used in Refs. [2,3].

**Figure 1:**
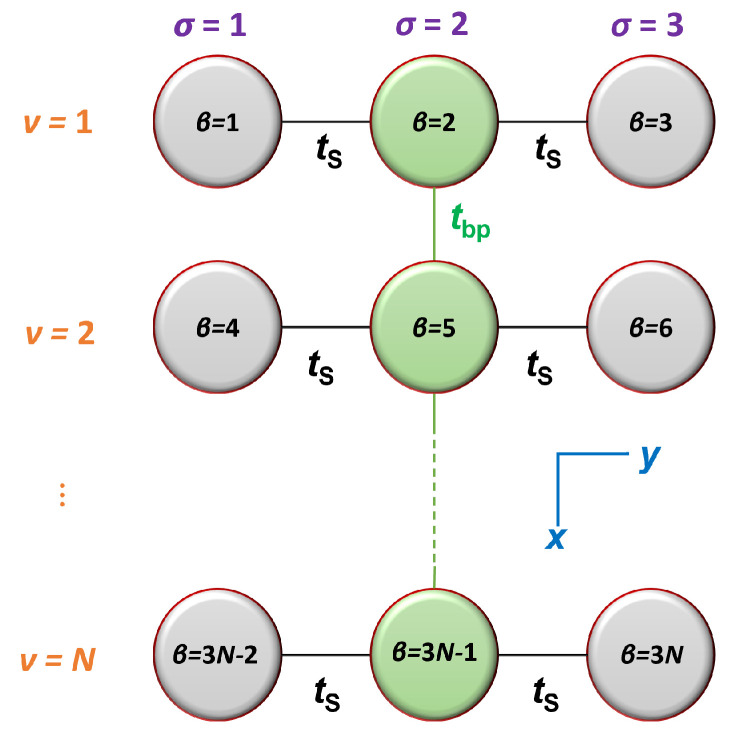
The FWM. σ=1,2,3 is the strand index, ν=1,2,…N is the row or monomer index, and β=1,2,…,3N is the site index. The relation between these indices is β=3(ν−1)+σ. In the absence of backbone disorder, all interaction integrals $t_{S}$ are equal, and all deoxyribose on-site energies $E_{S}$ are equal as well. The *x* and *y* axes are also indicated.

Fishbone-like models are named as such because the backbone sites are not connected to each other, yielding a fishbone shape (see Figure 1). Fishbone variants were used in Ref. [4] to study point mutation effects on charge transport in the tumor-suppressor gene p53, in Ref. [5] to analyze 162 disease-related genes, in Ref. [6] to study temperature and magnetic field effects on electron transport through DNA, among other models in Ref. [7], and in Ref. [8] to study the effect of phonons on electronic transport in DNA.

Additionally, a fishbone-like model was used in Ref. [9] to study the effects of disorder on the specific heat of DNA sequences, and an extended Fishbone-Ladder model was used in Ref. [10] to extract the current–voltage characteristics of DNA molecules oriented between two graphene nanoribbon electrodes. The FWM has also been employed to study the size-dependence [11] and spin-dependence [12] of electron transport along DNA homopolymers. Contrary to fishbone-like models, models that assume additional conduction through the backbone sites have also been proposed in the past, e.g., in Refs. [13,14,15,16].

The *hopping* integral or *transfer* integral or even *transport* integral or parameter are some of the terms used for quantities of the form $\langle \phi |\hat{H}|\psi \rangle$. These terms stem from the particular theory or problem examined, i.e., whether charge transfer or hopping transport or, generally, charge transport is studied. However, these quantities simply represent the interaction between states |ϕ〉 and |ψ〉 through the Hamiltonian $\hat{H}$. Therefore, a more generic term, not related to the particular problem or theory under investigation, would be the *interaction* integral or parameter—this is the term we are going to use throughout the text.

Even though the purine (guanine or adenine)–deoxyribose connection is not expected to be identical with the pyrimidine (cytosine or thymine)–deoxyribose connection, in this article, we make the simplification that the interaction integral between the base pairs and deoxyriboses, $t_{S}$, is identical from a base pair to the deoxyribose on the left or on the right.

The article is organized as follows: In Section 2, we describe the physical system and methods used, present the Hamiltonian in the second quantization picture, and discuss the time-independent problem to obtain the eigenstates and the density of states (DOS) and the time-dependent problem (the spatio-temporal evolution of a carrier created at a particular site). In Section 3, we present our results and discuss them; specifically, in Section 3.2, we discuss all ideal dimers and, in Section 3.1, the homopolymers. We examine the eigenspectra, DOS, and mean-over-time probabilities to find the carrier at each site, mean transfer rates between sites and the frequency content of carrier oscillations in the finite fishbone-wires under study. Finally, in Section 4, we state our conclusions.

## 2. System and Methods

A depiction of our FWM for DNA is provided in Figure 1. We consider a monomer to be a base pair together with its left and right deoxyriboses. Hence, each monomer is comprised of three sites: a base pair and two deoxyriboses connected to it. Between successive monomers, the only allowed interactions are the ones involving their respective base pairs; there are no interactions between the deoxyriboses of one monomer and any of the sites of its neighboring ones. We define three indices: σ=1,2,3 is the strand index, ν=1,2,…N is the row or monomer index, and β=1,2,…,3N is the site index. The relationship between these indices is

(1) β=3(ν−1)+σ.

We study all possible dimers and homopolymers. This work is a fishbone-wire extension of the wire model of Refs. [17,18], in the absence of backbone disorder. Each base pair has its own on-site energy, either $E_{G-C}$ or $E_{A-T}$. The interaction integral between successive base pairs is denoted by $t_{\mathrm{bp}}$. For base pairs, we use the parameterization of Ref. [18]. In the absence of backbone disorder, all interaction integrals, $t_{S}$, are equal, and all deoxyribose on-site energies, $E_{S}$, are equal as well.

### 2.1. Hamiltonian

The Hamiltonian describing the Highest Occupied Molecular Orbital (HOMO) or the Lowest Unoccupied Molecular Orbital (LUMO) regime of a given DNA polymer within the FWM is

(2) $$\begin{array}{cc} \hat{H}= & \sum_{\nu =1}^{N}E_{\nu \;2}|\nu \;2\rangle \langle \nu \;2|+\sum_{\nu =1}^{N-1}t_{\nu \;2,\nu \;+\;1\;2}|\nu \;2\rangle \langle \nu \;+\;1\;2|+\sum_{\nu =2}^{N}t_{\nu \;2,\nu \;-\;1\;2}|\nu \;2\rangle \langle \nu \;-\;1\;2|\\ + & \sum_{\nu =1}^{N}E_{\nu \;1}|\nu \;1\rangle \langle \nu \;1|+\sum_{\nu =1}^{N}t_{\nu \;2,\nu \;1}|\nu \;2\rangle \langle \nu \;1|+\sum_{\nu =1}^{N}t_{\nu \;1,\nu \;2}|\nu \;1\rangle \langle \nu \;2|\\ + & \sum_{\nu =1}^{N}E_{\nu \;3}|\nu \;3\rangle \langle \nu \;3|+\sum_{\nu =1}^{N}t_{\nu \;2,\nu \;;3}|\nu \;2\rangle \langle \nu \;3|+\sum_{\nu =1}^{N}t_{\nu \;3,\nu \;2}|\nu \;3\rangle \langle \nu \;2|, \end{array}$$

where $E_{\nu \;\sigma },t_{\nu \;\sigma ,\nu^{\prime }\;\sigma^{\prime }}$ are the relevant on-site energies and interaction parameters, respectively. To simplify the notation, we omit HOMO (or LUMO) from the names of all symbols. Using, e.g., the β index, we can represent the above Hamiltonian as a 3N×3N matrix, *H*. For, example, the Hamiltonian matrix of a dimer has the form

(3) $$H=\left[\begin{array}{cccccc} E_{S} & t_{S} & 0 & 0 & 0 & 0\\ t_{S} & E_{\mathrm{bp}1} & t_{S} & 0 & t_{12} & 0\\ 0 & t_{S} & E_{S} & 0 & 0 & 0\\ 0 & 0 & 0 & E_{S} & t_{S} & 0\\ 0 & t_{21} & 0 & t_{S} & E_{\mathrm{bp}2} & t_{S}\\ 0 & 0 & 0 & 0 & t_{S} & E_{S} \end{array}\right].$$

For base pairs, we denote the on-site energy of base pair *i* by $E_{\mathrm{bp}i}$ and the interaction integral between base pairs *i* and *j* by $t_{ij}$. For deoxyriboses (sugars), the on-site energy is $E_{S}$, and the interaction integral between base pairs and deoxyriboses is $t_{S}$. In this work, we keep $E_{S}$ and $t_{S}$ constant, i.e., we ignore backbone disorder either in $E_{S}$ or in $t_{S}$. Effects in the transmission of the FWM, assuming different values at the backbone on-site energies at either side of the G-C base pair were studied in Ref. [19].

### 2.2. Time-Independent Problem

The state, |ψ〉, of the macromolecule can be written as a linear combination of the deoxyribose states on the left of base pairs, |ν1〉, the base pair states, |ν2〉, and the deoxyribose states on the right of base pairs, |ν3〉, with time-independent coefficients $l_{\nu }$, $a_{\nu }$, and $r_{\nu }$, respectively, i.e.,

(4) $$|\psi \rangle =\sum_{\nu =1}^{N}l_{\nu }|\nu \;1\rangle +a_{\nu }|\nu \;2\rangle +r_{\nu }|\nu \;3\rangle .$$

Plugging Equation (4) into the time-independent Schrödinger equation,

(5) $$\hat{H}|\psi \rangle =E|\psi \rangle ,$$

we obtain

(6) $$\sum_{\nu =1}^{N}l_{\nu }\hat{H}|\nu \;1\rangle +a_{\nu }\hat{H}|\nu \;2\rangle +r_{\nu }\hat{H}|\nu \;3\rangle =E\sum_{\nu =1}^{N}l_{\nu }|\nu \;1\rangle +a_{\nu }|\nu \;2\rangle +r_{\nu }|\nu \;3\rangle .$$

Multiplying Equation (6) by 〈λ1| yields

(7) $$\begin{array}{c} \sum_{\nu =1}^{N}l_{\nu }\langle \lambda \;1|\hat{H}|\nu \;1\rangle +a_{\nu }\langle \lambda \;1|\hat{H}|\nu \;2\rangle +r_{\nu }\langle \lambda \;1|\hat{H}|\nu \;3\rangle =\\ E\sum_{\nu =1}^{N}l_{\nu }\langle \lambda \;1|\nu \;1\rangle +a_{\nu }\langle \lambda \;1|\nu \;2\rangle +r_{\nu }\langle \lambda \;1|\nu \;3\rangle . \end{array}$$

Given that, within the TB approximation for the FWM, $\langle \lambda \;1|\nu \;1\rangle =\delta_{\lambda \nu }$, 〈λ1|ν2〉=0, 〈λ1|ν3〉=0, and $\langle \lambda \;1|\hat{H}|\nu \;1\rangle =\delta_{\lambda \nu }E_{S}$, $\langle \lambda \;1|\hat{H}|\nu \;2\rangle =\delta_{\lambda \nu }t_{S}$, $\langle \lambda \;1|\hat{H}|\nu \;3\rangle =0$, we arrive at

(8) $$l_{\lambda }E_{S}+a_{\lambda }t_{S}=El_{\lambda }.$$

Similarly, multiplying Equation (6) by 〈λ2| yields

(9) $$\begin{array}{c} \sum_{\nu =1}^{N}l_{\nu }\langle \lambda \;2|\hat{H}|\nu \;1\rangle +a_{\nu }\langle \lambda \;2|\hat{H}|\nu \;2\rangle +r_{\nu }\langle \lambda \;2|\hat{H}|\nu \;3\rangle =\\ E\sum_{\nu =1}^{N}l_{\nu }\langle \lambda \;2|\nu \;1\rangle +a_{\nu }\langle \lambda \;2|\nu \;2\rangle +r_{\nu }\langle \lambda \;2|\nu \;3\rangle . \end{array}$$

Given that 〈λ2|ν1〉=0, $\langle \lambda \;2|\nu \;2\rangle =\delta_{\lambda \nu }$, 〈λ2|ν3〉=0, and $\langle \lambda \;2|\hat{H}|\nu \;1\rangle =\delta_{\lambda \nu }t_{S}$, $\langle \lambda \;2|\hat{H}|\nu \;2\rangle =\delta_{\lambda \nu }E_{\mathrm{bp}}$, $\langle \lambda \;2|\hat{H}|\nu \;3\rangle =\delta_{\lambda \nu }t_{S}$, $\langle \lambda \;2|\hat{H}|\nu \;+\;1\;2\rangle =\delta_{\lambda \nu }t_{\lambda ,\nu \;+\;1}$, $\langle \lambda \;2|\hat{H}|\nu \;-\;1\;2\rangle =\delta_{\lambda \nu }t_{\lambda ,\nu \;-\;1}$, we arrive at

(10) $$l_{\lambda }t_{S}+a_{\lambda -1}t_{\lambda ,\lambda \;-\;1}+a_{\lambda }E_{\mathrm{bp}}+a_{\lambda +1}t_{\lambda ,\lambda \;+\;1}+r_{\lambda }t_{S}=Ea_{\lambda }.$$

Finally, multiplying Equation (6) by 〈λ3| yields

(11) $$\begin{array}{c} \sum_{\nu =1}^{N}l_{\nu }\langle \lambda \;3|\hat{H}|\nu \;1\rangle +a_{\nu }\langle \lambda \;3|\hat{H}|\nu \;2\rangle +r_{\nu }\langle \lambda \;3|\hat{H}|\nu \;3\rangle =\\ \sum_{\nu =1}^{N}l_{\nu }\langle \lambda \;3|\nu \;1\rangle +a_{\nu }\langle \lambda \;3|\nu \;2\rangle +r_{\nu }\langle \lambda \;3|\nu \;3\rangle . \end{array}$$

Given that 〈λ3|ν1〉=0, 〈λ3|ν2〉=0, $\langle \lambda \;3|\nu \;3\rangle =\delta_{\lambda \nu }$, and $\langle \lambda \;3|\hat{H}|\nu \;1\rangle =0$, $\langle \lambda \;3|\hat{H}|\nu \;2\rangle =\delta_{\lambda \nu }t_{S}$, $\langle \lambda \;3|\hat{H}|\nu \;3\rangle =\delta_{\lambda \nu }E_{S}$, we arrive at

(12) $$a_{\lambda }t_{S}+r_{\lambda }E_{S}=Er_{\lambda }.$$

For example, for a dimer (λ=1,2), using Equations (8), (10) and (12), we obtain

$$\begin{array}{cc} l_{1}E_{S}+a_{1}t_{S} & =El_{1}\\ l_{1}t_{S}+a_{1}E_{1}+r_{1}t_{S}+a_{2}t_{12} & =Ea_{1}\\ a_{1}t_{S}+r_{1}E_{S} & =Er_{1}\\ l_{2}E_{S}+a_{2}t_{S} & =El_{2}\\ a_{1}t_{21}+l_{2}t_{S}+a_{2}E_{2}+r_{2}t_{S} & =Ea_{2}\\ a_{2}t_{S}+r_{2}E_{S} & =Er_{2}, \end{array}$$

which, in matrix form, is [cf. Equation (2)]

(13) $$\left[\begin{array}{cccccc} E_{S} & t_{S} & 0 & 0 & 0 & 0\\ t_{S} & E_{1} & t_{S} & 0 & t_{12} & 0\\ 0 & t_{S} & E_{S} & 0 & 0 & 0\\ 0 & 0 & 0 & E_{S} & t_{S} & 0\\ 0 & t_{21} & 0 & t_{S} & E_{2} & t_{S}\\ 0 & 0 & 0 & 0 & t_{S} & E_{S} \end{array}\right]\left[\begin{array}{c} l_{1}\\ a_{1}\\ r_{1}\\ l_{2}\\ a_{2}\\ r_{2} \end{array}\right]=E\left[\begin{array}{c} l_{1}\\ a_{1}\\ r_{1}\\ l_{2}\\ a_{2}\\ r_{2} \end{array}\right].$$

The time-independent problem expressed in Equations (8), (10), and (12) can be written in the general form

(14) $$H\vec{v}=E\vec{v},$$

where *H* is the Hamiltonian matrix and $\vec{v}$ is a vector with 3N components, i.e.,

(15) $$\vec{v}=\left[\begin{array}{c} l_{1}\\ a_{1}\\ r_{1}\\ \vdots \\ l_{N}\\ a_{N}\\ r_{N} \end{array}\right].$$

In other words, we have to solve an eigenvalue–eigenvector problem.

### 2.3. Density of States

The DOS, g(E), is the number of eigenenergies, $dN_{E}$, in the energy interval (*E*, E+dE). It can be calculated through the eigenenergies $E_{k}$, where *k* is a collective generic eigenenergy index. In our FWM, for *N* monomers, we have 3N eigenenergies. Hence,

(16) $$g\left(E\right)=\sum_{k=1}^{3N}\delta \left(E-E_{k}\right).$$

For a small number of monomers, *N*, discrete energy levels occur. However, as *N* increases, the eigenenergies are gathered into subbands. The DOS diagrams demonstrated in Section 3 are obtained for large *N* (=2000). We notice that the persistence length of DNA is ≈150 monomers, and so our quasi one-dimensional model is not really adequate for longer distances. Additionally, we are dealing with coherent phenomena, which are expected to dominate at small distances. The main reason for using large *N* for DOS diagrams is to obtain smooth images. The number of subbands is equal to the number of sites within the repetition unit. For homopolymers, a monomer (which is also the repetition unit) is composed of three sites; hence, three subbands are obtained. The states are distributed in energy regions close to the on-site energies of base pairs (A–T or G–C) as well as to the deoxyribose on-site energy.

### 2.4. Time-Dependent Problem

The state, |ψ(t)〉, of the studied macromolecule can be written as a linear combination of the deoxyribose states on the left of base pairs, |ν1〉; the base pair states, |ν2〉; and the deoxyribose states on the right of base pairs, |ν3〉, with relevant time-dependent coefficients $L_{\nu }\left(t\right)$, $A_{\nu }\left(t\right)$, $R_{\nu }\left(t\right)$, i.e.,

(17) $$|\psi (t)\rangle =\sum_{\nu =1}^{N}L_{\nu }\left(t\right)|\nu \;1\rangle +A_{\nu }\left(t\right)|\nu \;2\rangle +R_{\nu }\left(t\right)|\nu \;3\rangle .$$

Plugging Equation (17) into the time-dependent Schrödinger equation,

(18) $$i\hbar \frac{\partial |\psi (t)\rangle }{\partial t}=\hat{H}|\psi (t)\rangle ,$$

we obtain

(19) $$\begin{array}{c} i\hbar \sum_{\nu =1}^{N}{\dot{L}}_{\nu }\left(t\right)|\nu \;1\rangle +{\dot{A}}_{\nu }\left(t\right)|\nu \;2\rangle +{\dot{R}}_{\nu }\left(t\right)|\nu \;3\rangle =\\ \sum_{\nu =1}^{N}L_{\nu }\left(t\right)\hat{H}|\nu \;1\rangle +A_{\nu }\left(t\right)\hat{H}|\nu \;2\rangle +R_{\nu }\left(t\right)\hat{H}|\nu \;3\rangle . \end{array}$$

Multiplying Equation (19) by 〈λ1| yields

(20) $$\begin{array}{c} i\hbar \sum_{\nu =1}^{N}{\dot{L}}_{\nu }\left(t\right)\langle \lambda \;1|\nu \;1\rangle +{\dot{A}}_{\nu }\left(t\right)\langle \lambda \;1|\nu \;2\rangle +{\dot{R}}_{\nu }\left(t\right)\langle \lambda \;1|\nu \;3\rangle =\\ \sum_{\nu =1}^{N}L_{\nu }\left(t\right)\langle \lambda \;1|\hat{H}|\nu \;1\rangle +A_{\nu }\left(t\right)\langle \lambda \;1|\hat{H}|\nu \;2\rangle +R_{\nu }\left(t\right)\langle \lambda \;1|\hat{H}|\nu \;3\rangle , \end{array}$$

and, since, within the TB approximation for the FWM, $\langle \lambda \;1|\nu \;1\rangle =\delta_{\lambda \nu }$, 〈λ1|ν2〉=0, 〈λ1|ν3〉=0, and $\langle \lambda \;1|\hat{H}|\nu \;1\rangle =\delta_{\lambda \nu }E_{S}$, $\langle \lambda \;1|\hat{H}|\nu \;2\rangle =\delta_{\lambda \nu }t_{S}$, $\langle \lambda \;1|\hat{H}|\nu \;3\rangle =0$, we arrive at

(21) $$i\hbar {\dot{L}}_{\lambda }\left(t\right)=L_{\lambda }\left(t\right)E_{S}+A_{\lambda }\left(t\right)t_{S}.$$

Similarly, multiplying Equation (19) by 〈λ2| yields

(22) $$\begin{array}{c} i\hbar \sum_{\nu =1}^{N}{\dot{L}}_{\nu }\left(t\right)\langle \lambda \;2|\nu \;1\rangle +{\dot{A}}_{\nu }\left(t\right)\langle \lambda \;2|\nu \;2\rangle +{\dot{R}}_{\nu }\left(t\right)\langle \lambda \;2|\nu \;3\rangle =\\ \sum_{\nu =1}^{N}L_{\nu }\left(t\right)\langle \lambda \;2|\hat{H}|\nu \;1\rangle +A_{\nu }\left(t\right)\langle \lambda \;2|\hat{H}|\nu \;2\rangle +R_{\nu }\left(t\right)\langle \lambda \;2|\hat{H}|\nu \;3\rangle , \end{array}$$

and, since 〈λ2|ν1〉=0, $\langle \lambda \;2|\nu \;2\rangle =\delta_{\lambda \nu }$, 〈λ2|ν3〉=0, $\langle \lambda \;2|\hat{H}|\nu \;1\rangle =\delta_{\lambda \nu }t_{S}$, $\langle \lambda \;2|\hat{H}|\nu \;2\rangle =\delta_{\lambda \nu }E_{S}$, $\langle \lambda \;1|\hat{H}|\nu \;3\rangle =0$, we arrive at

(23) $$i\hbar {\dot{A}}_{\lambda }\left(t\right)=L_{\lambda }\left(t\right)t_{S}+A_{\lambda }\left(t\right)E_{\lambda }+A_{\lambda +1}\left(t\right)t_{\lambda ,\lambda +1}+A_{\lambda -1}\left(t\right)t_{\lambda ,\lambda -1}+R_{\lambda }\left(t\right)t_{S}.$$

Finally, multiplying Equation (19) by 〈λ3| yields

(24) $$\begin{array}{c} i\hbar \sum_{\nu =1}^{N}{\dot{L}}_{\nu }\left(t\right)+\langle \lambda \;3|\nu \;1\rangle +{\dot{A}}_{\nu }\left(t\right)\langle \lambda \;3|\nu \;2\rangle +{\dot{R}}_{\nu }\left(t\right)\langle \lambda \;3|\nu \;3\rangle =\\ \sum_{\nu =1}^{N}L_{\nu }\left(t\right)\langle \lambda \;3|\hat{H}|\nu \;1\rangle +A_{\nu }\left(t\right)\langle \lambda \;3|\hat{H}|\nu \;2\rangle +R_{\nu }\left(t\right)\langle \lambda \;3|\hat{H}|\nu \;3\rangle , \end{array}$$

and, since 〈λ3|ν1〉=0, 〈λ3|ν2〉=0, $\langle \lambda \;3|\nu \;3\rangle =\delta_{\lambda \nu }$ and $\langle \lambda \;3|\hat{H}|\nu \;1\rangle =0$, $\langle \lambda \;3|\hat{H}|\nu \;2\rangle =\delta_{\lambda \nu }t_{S}$, $\langle \lambda \;3|\hat{H}|\nu \;3\rangle =\delta_{\lambda \nu }E_{S}$, we arrive at

(25) $$i\hbar {\dot{R}}_{\lambda }\left(t\right)=A_{\lambda }\left(t\right)t_{S}+R_{\lambda }\left(t\right)E_{S}.$$

The time-dependent problem expressed in Equations (21), (23), and (25), can generally be written in the form of a first-order matrix differential equation,

(26) $$\dot{\vec{C}}\left(t\right)=-\frac{i}{\hbar }H\vec{C}\left(t\right),$$

where

(27) $$\vec{C}\left(t\right)=\left[\begin{array}{c} L_{1}\left(t\right)\\ A_{1}\left(t\right)\\ R_{1}\left(t\right)\\ \vdots \\ L_{N}\left(t\right)\\ A_{N}\left(t\right)\\ R_{N}\left(t\right) \end{array}\right].$$

$\vec{C}\left(t\right)$ has 3N components. *H* is the Hamiltonian matrix. Equation (26) can be solved with the eigenvalue method, i.e., by looking for solutions of the form $\vec{C}\left(t\right)=\vec{u}e^{-\frac{i}{\hbar }\varepsilon t}\Rightarrow \dot{\vec{C}}\left(t\right)=-\frac{i}{\hbar }\varepsilon \vec{u}e^{-\frac{i}{\hbar }\varepsilon t}$. Hence, Equation (26) leads to $H\vec{u}=\varepsilon \vec{u}$, i.e., to the eigenvalue problem of Equation (14), i.e., $\vec{u}$ are the eigenvectors $\vec{v}$ and ε are the eigenvalues *E*. Having determined the eigenvalues and eigenvectors of *H*, the general solution of Equation (26) is

(28) $$\vec{C}\left(t\right)=\sum_{k=1}^{3N}C_{k}{\vec{v}}_{k}e^{-\frac{i}{\hbar }E_{k}t},$$

where the coefficients $C_{k}$ are determined by the initial conditions. In particular, if we define the 3N×3N eigenvector matrix *V*, with elements $v_{jk}$ (*j*-th component of *k*-th eigenvector), then it can be shown that the vector matrix $\vec{C}$, composed of the coefficients $C_{k},\;\;k=1,2,\ldots ,3N$, is given by the expression

(29) $$\vec{C}=V^{T}\vec{C}\left(0\right).$$

Let us now suppose that, initially, the extra carrier is placed at site α, i.e., $C_{\alpha }\left(0\right)=1$, $C_{\beta }\left(0\right)=0,\forall \beta \neq \alpha$. Then,

(30) $$\vec{C}=\left[\begin{array}{c} v_{\alpha 1}\\ \vdots \\ v_{\alpha k}\\ \vdots \\ v_{\alpha N} \end{array}\right].$$

In other words, the coefficients $C_{k}$ are given by the row of the eigenvector matrix, which corresponds to the site the carrier is initially placed at. In this work, we choose α=2 or 3, i.e., we initially place the carrier (a hole, under the assumption that holes travel through HOMOs) at the first monomer, either at the base pair (α=2) or at the deoxyribose on its right (α=3), unless otherwise stated.

From Equation (28), it follows that the probability to find the extra carrier at the β-th site is

(31) $$|C_{\beta }{\left(t\right)|}^{2}=\sum_{k=1}^{3N}C_{k}^{2}v_{\beta k}^{2}+2\sum_{k=1}^{3N}\sum_{\begin{array}{c} k^{\prime }=1\\ k^{\prime }<k \end{array}}^{3N}C_{k}C_{k^{\prime }}v_{\beta k}v_{\beta k^{\prime }}\cos\left(2\pi f_{kk^{\prime }}t\right),$$

where

(32) $$f_{kk^{\prime }}=\frac{1}{T_{kk^{\prime }}}=\frac{E_{k}-E_{k^{\prime }}}{h},\;\forall k>k^{\prime },$$

are the frequencies ($f_{kk^{\prime }}$) or periods ($T_{kk^{\prime }}$) involved in charge transfer.

If the number of discrete eigenenergies is *m*, then the number of different $f_{kk^{\prime }}$ or $T_{kk^{\prime }}$ involved in carrier transfer is S=m2=m!2!(m−2)!=m(m−1)2. Additionally, degenerate eigenenergies result in a zero frequency (infinite period). If there were no degenerate eigenenergies, then *m* would be 3N. However, multiple degeneracies exist for ideal homopolymers in the FWM, unless diagonal or off-diagonal disorder is included. In the absence of disorder and considering $t_{S}$ as having the same value for both connections of the base pair to its left and right deoxyriboses, if $t_{S}=0$, then there are 2N degenerate eigenvalues, equal to $E_{S}$. If $t_{S}\neq 0$, then there are *N* degenerate eigenvalues equal to $E_{S}$ as will be shown below in more detail. Another case that exhibits degeneracies is, e.g., *cyclic* homopolymers within the wire model [20].

From Equation (31), it follows that, if there were no deneneracies, and for real $C_{k}$, $v_{\beta k}$, the mean-over-time probability to find the carrier at the β-th site would be $\langle |C_{\beta }\left(t\right){|}^{2}\rangle =\sum_{k=1}^{3N}C_{k}^{2}v_{\beta k}^{2}.$ However, for homopolymers in the FWM without disorder, multiple degeneracies exist; hence, $\langle |C_{\beta }\left(t\right){|}^{2}\rangle$ have to be calculated directly from Equation (31), i.e., by numerically averaging $|C_{\beta }{\left(t\right)|}^{2}$ over time.

Furthermore, from Equation (31), it can be shown that, as in Ref. [21], the one-sided Fourier amplitude spectrum that corresponds to the probability $|C_{\beta }{\left(t\right)|}^{2}$ is given by

(33) $$|F_{\beta }\left(f\right)|=\sum_{k=1}^{3N}C_{k}^{2}v_{\beta k}^{2}\delta \left(f\right)+2\sum_{k=1}^{3N}\sum_{\begin{array}{c} k^{\prime }=1\\ k^{\prime }<k \end{array}}^{3N}\left|C_{k}C_{k^{\prime }}v_{\beta k}v_{\beta k^{\prime }}\right|\delta \left(f-f_{kk^{\prime }}\right).$$

Hence, the Fourier amplitude of frequency $f_{kk^{\prime }}$ is $2|C_{k}v_{\beta k}C_{k^{\prime }}v_{\beta k^{\prime }}|$. We can further define the Weighted Mean Frequency (WMF) of site β as

(34) $$f_{WM\beta }=\frac{\sum_{k=1}^{3N}\sum_{\begin{array}{c} k^{\prime }=1\\ k^{\prime }<k \end{array}}^{3N}\left|C_{k}v_{\beta k}C_{k^{\prime }}v_{\beta k^{\prime }}\right|f_{kk^{\prime }}}{\sum_{k=1}^{3N}\sum_{\begin{array}{c} k^{\prime }=1\\ k^{\prime }<k \end{array}}^{3N}\left|C_{k}v_{\beta k}C_{k^{\prime }}v_{\beta k^{\prime }}\right|}.$$

WMF expresses the mean frequency content of the carrier oscillation at site β. Having determined the WMF for all sites, it is possible to obtain a measure of the overall frequency content of the carrier oscillation in the system. Since $f_{WM\beta }$ is the WMF of site β and $\langle |C_{\beta }{\left(t\right)|}^{2}\rangle$ is the mean probability of finding the carrier at site β, we define the total weighted mean frequency (TWMF) as

(35) $$f_{TWM}=\sum_{\beta =1}^{3N}f_{WM\beta }\langle |C_{\beta }{\left(t\right)|}^{2}\rangle .$$

The mean transfer rate, $k_{\alpha \beta }$, expresses the rate at which a carrier is transferred from site α to site β. It is a quantity defined for coherent charge transfer, and it takes into account both the amount of probability transfer and the time-scale of transfer [18]. For initial placement at site α, it is defined as

(36) $$k_{\alpha \beta }=\frac{\langle |C_{\beta }{\left(t\right)|}^{2}\rangle }{t_{\alpha \beta }},$$

where $t_{\alpha \beta }$ is the time at which the time-dependent probability to find the carrier at site β, $|C_{\beta }{\left(t\right)}^{2}|$, becomes equal to its mean-over-time value, $\langle |C_{\beta }{\left(t\right)}^{2}|\rangle$, for the first time.

The evolution of the probability to find the extra carrier at each site reflects the character of the charge movement within the polymer. Another useful relevant quantity is the time-evolution of the dipole moment relative to the center of the molecule. Within the FWM, we define the *x*-axis along the molecule and the *y*-axis across it (cf. Figure 1). Moving along the *x*-axis, the monomer index ν changes; moving along the *y*-axis, the strand index σ changes. The distance between successive monomers (adjacent strands) is taken as $d_{x}=3.4$ Å ($d_{y}=10$ Å). Hence, the *x*- and *y*-components of the dipole moment are

(37) $$\begin{array}{c} P_{x}=\pm ed_{x}\sum_{\beta =1}^{3N}\left(\nu -\nu \left(x_{c}\right)\right){\left|C_{\beta }\left(t\right)\right|}^{2} \end{array}$$

(38) $$\begin{array}{c} P_{y}=\pm ed_{y}\sum_{\beta =1}^{3N}\left(\sigma -\sigma \left(y_{c}\right)\right){\left|C_{\beta }\left(t\right)\right|}^{2}, \end{array}$$

where β, ν, and σ are connected to each other via Equation (1), $x_{c}$ and $y_{c}$ specify the center of the molecule, *e* is the elementary charge, and ± denotes different signs for electron or hole transfer.

## 3. Results and Discussion

We note that, when dealing with hole transfer, the electronic level energies and the respective interaction integrals have to be used with opposite signs since a hole represents a positive charge. In this work, the deoxyribose on-site energy is considered $E_{S}=9.0$ eV. This value is taken from Ref. [22], i.e., it is equal to the ionization energy of gas-phase deoxyribose and opposite to the corresponding HOMO electronic level energy. The value of $E_{S}$ may vary since different bases are connected to deoxyriboses at each strand; it will also be affected by the phosphate group connected to each deoxyribose and the presence of water molecules and counterions attached to the backbone [23].

However, the value 12.27 eV proposed in Ref. [23] seems to be far from the ionization energy of deoxyribose. In Refs. [15,16], the backbone on-site energies of 8.85 eV were assumed, but those models allowed also for conduction between backbone sites. In the extended Fishbone-Ladder Model of Ref. [10], the backbone on-site energy was assumed to be 8.5 eV for all backbone sites. Assuming $E_{S}=9.0$ eV, i.e., a value close to the on-site energies of bases or base pairs (which lie in the region of 8 to 9 eV [17]) leads to a strong interaction of the backbone with the stack of base pairs, e.g., see Section 3.1.1 and Section 3.1.2.

Regarding the value of the interaction parameter between base pairs and deoxyriboses, $t_{S}$, different, more or less arbitrary values have been assumed in the literature, e.g., 1.5 [23], 1.0 [24], 0.7 [9], and 0.74 or 0.24 eV [2]. Therefore, in this work, we explore the influence of different values of $t_{S}$. The sign of $t_{S}$ does not affect the eigenspectra. The base pair on-site energies and interaction parameters between base pairs were taken from the parametrization of Ref. [18], which considered the previous works [17,25,26,27,28,29]. The on-site energies of the two possible base pairs are $E_{G-C}=8$ eV and $E_{A-T}=8.3$ eV, i.e., opposite to the corresponding HOMO electronic level energies [17]. We use the interaction integrals between consecutive base pairs (or *dimers*) that are shown in Table 1, which are also taken with opposite signs to the calculated couplings between the HOMO electronic levels.

Structural variability plays a significant role in modifying the values of interaction integrals, but the charge transfer in homodimers remains, on average, significant [30,31]. However, such effects are not considered in the present work, which is focused on a general overview of the FWM for homopolymers and dimers. There are three categories of DNA dimers: (a) Made of the same monomer with purine on purine (and pyrimidine on pyrimidine), i.e., GG≡CC and AA≡TT. (b) Made of the same monomer with purine on pyrimidine, i.e., GC, CG, AT, and TA. (c) Made of different monomers, i.e., AG≡CT, TC≡GA, AC≡GT, and TG≡CA.

The notation XY means that dimers are named using their bases in the 5${}^{\prime }$–3${}^{\prime }$ direction. Hence, e.g., TG≡CA, since the sequence TG in the 5${}^{\prime }$–3${}^{\prime }$ direction of one strand gives the same dimer with the sequence CA in the 5${}^{\prime }$–3${}^{\prime }$ direction of the complementary strand.

### 3.1. Homopolymers

Our results are presented as follows: In Section 3.1.1, we discuss the eigenspectra of homooligomers, i.e., systems composed of a small number of identical monomers with purines on purines. All results refer to G... ≡ C... oligomers or homopolymers. The results for A... ≡ T... sequences are, of course, qualitatively identical. Then, for illustrative purposes, we proceed to the large-*N* regime (ideally to N→∞) and present the corresponding DOS in Section 3.1.2.

The above quantities are relevant to the time-independent problem. Next, we proceed to quantities related to the time-dependent problem of oxidizing one site (either base pair or deoxyribose) and tracking the temporal and spatial evolution of the created hole. In Section 3.1.3, we discuss the mean amount of charge at all sites, i.e., the mean-over-time probabilities. In Section 3.1.4, we discuss the frequency content of carrier oscillations within our system (standing waves within the fishbone). Having already discussed the amount of charge and the frequency content, in Section 3.1.5, we present the coherent mean transfer rates, which take into account both the amount of transferred charge and the temporal scale of the phenomenon.

#### 3.1.1. Eigenspectra

**Figure 2:**
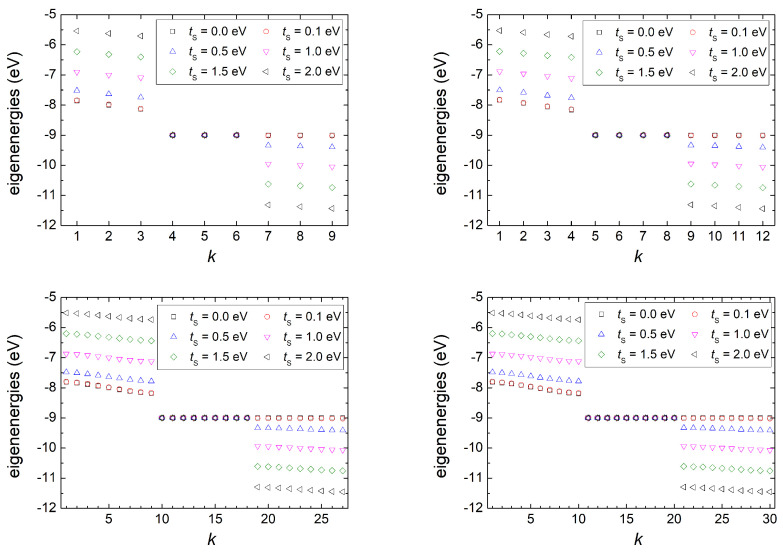
The eigenenergies, indexed in descending order by *k* for different values of the interaction parameter between base pairs and deoxyriboses, $t_{S}$ for a G... homopolymer made of N= 3, 4, 9, and 10 monomers (from top left to bottom right).

In Figure 2, we compare the eigenenergies of G... homopolymers made of N=3, 4, 9, 10 G monomers for five different values of the interaction parameter between base pairs and deoxyriboses, $t_{S}$. For $t_{S}=0$, the base pairs do not interact with deoxyriboses; hence, the FWM collapses to the wire model plus isolated deoxyriboses. 2N eigenenergies are identical, equal to the on-site energies of isolated deoxyriboses, $E_{S}$. The remaining *N* eigenenergies are the ones predicted from the wire model for DNA base pairs [20] according to the parametrization of Ref. [18]. In total, we have a single subband from the base pairs, identical to the wire model, and a degenerate subband from deoxyriboses at $E_{S}$.

Switching the interaction between base pairs and deoxyriboses on (i.e., for $t_{S}\neq 0$) and gradually increasing its strength, i.e., for $t_{S}=$ 0.1, 0.5, 1.0, 1.5, and 2.0 eV, we observe that half of the 2N degeneracies of the deoxyribose subband are lifted, and three subbands are formed in total: one comprised of *N* degenerate ($E_{S}=9.0$ eV) deoxyribose eigenvalues, and two comprised of *N* eigenvalues each, above and below $E_{S}$. It is worth noting that these two (upper and lower) subbands are not symmetrically positioned around the central one ($E_{S}$) and that, as $t_{S}$ increases, the two non-degenerate subbands move further away from each other and from the degenerate one.

From a glance at Figure 2, it seems that the eigenenergies in each subband are symmetrically positioned relative to the subband’s center. However, this is not exactly true: The central eigenenergies of each subband are more symmetrically positioned relative to its center than peripheral eigenenergies. This effect is enhanced, increasing *N*. This is at odds with the wire model, within which the homopolymer eigenenergies (corresponding to the upper subbands of this FWM) are symmetrically positioned around their center [20].

In Ref. [32], the authors showed similar dispersion curves for a particular case, but they *removed* the eigenenergies close to $E_{S}$ due to singular behavior of their equations at this energy; a limitation of the FWM already implied in Ref. [1]. On the contrary, in Ref. [33], where thermoelectric transport in the homopolymer G... was studied, the eigenenergies at $E_{S}$ were mirrored in the electronic transmission curves.

#### 3.1.2. Density of States

**Figure 3:**
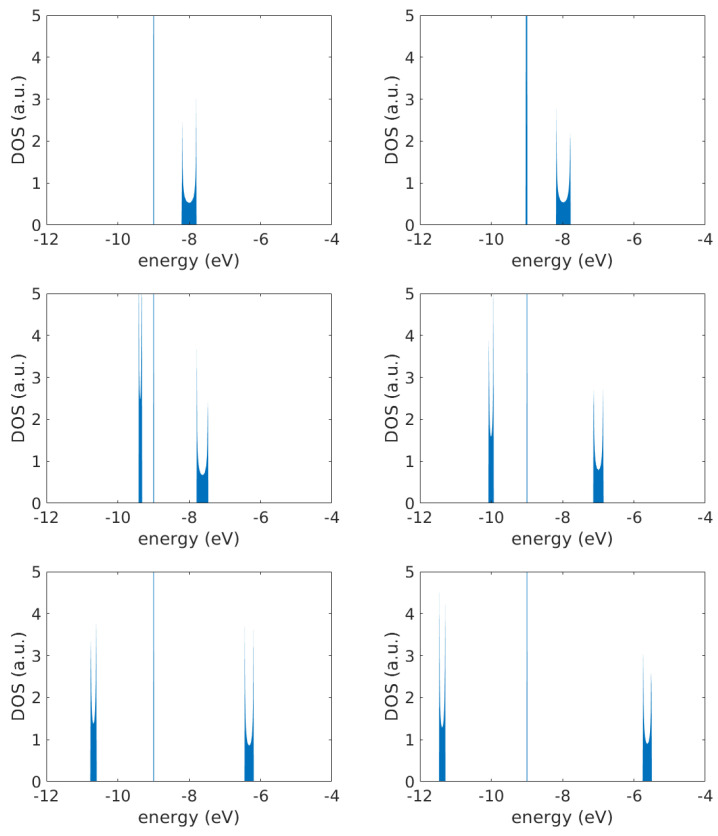
FWM DOS for a G... homopolymer with N=2000 for $t_{S}=$ 0.0, 0.1, 0.5, 1.0, 1.5, and 2.0 eV (from top left to bottom right). In an infinite system, there are van Hove singularities at the subband edges, and the central degenerate band at $E_{S}$ becomes infinitely high.

Isolated deoxyriboses have 2N eigenenergies, and isolated base pairs have *N* eigenenergies. As shown in Section 3.1.1 and depicted in Figure 2, when they interact, we obtain 3N eigenenergies, divided into three subbands. Figure 3 depicts the DOS of a G... homopolymer composed of a large number of monomers (N=2000) for varying $t_{S}$. The diagrams were obtained by counting the eigenenergies derived by numerically diagonalizing the Hamiltonian matrix at appropriately chosen energy intervals. The very thin (in fact, degenerate) subband positioned at $E_{S}$ is comprised of *N* eigenenergies. The subband at larger energies is comprised of *N* eigenenergies, as well; it corresponds to the base-pair band predicted from the wire model [20] but repulsed from $E_{S}$ due to the interaction between base pairs and deoxyriboses ($t_{S}$).

The subband at lower energies is also comprised of *N* eigenenergies; it originates from deoxyriboses but is repulsed due to the interaction with the base pairs. It is clear in Figure 3 that, increasing $t_{S}$, the subbands move further away from each other, and their width gradually changes. Specifically, for $t_{S}=0$, as expected from Ref. [21], the base-pair wire bandwidth is $4|t_{\mathrm{GG}}|$, i.e., 0.4 eV for our parametrization. For $t_{S}=0.5$ eV, the widths are ≈0.08 eV for the left subband and ≈0.31 eV for the right one. For $t_{S}=1$ eV, the widths are ≈0.13 eV for the left subband and ≈0.27 eV for the right one. Therefore, increasing $t_{S}$ increases the width of the non-degenerate deoxyribose subband and, at the same time, decreases the width of the base-pair subband. In an infinite system, van Hove singularities at the subband edges occur, and the central degenerate band at $E_{S}$ becomes infinitely high.

#### 3.1.3. Mean-over-Time Probabilities

We are now interested in the influence of increasing $t_{S}$ on the mean-over-time probability to find the carrier at each site, i.e., on $\langle |C_{\beta }\left(t\right){|}^{2}\rangle ,\forall \beta$. Let us denote the site where the hole is initially placed by α. Hence, with the exception of site α, $\langle |C_{\beta }\left(t\right){|}^{2}\rangle$ is equal to the carrier transfer percentage from site α to site β. For site α, $\langle |C_{\alpha }\left(t\right){|}^{2}\rangle$ is the carrier percentage that is not transferred.

**Figure 4:**
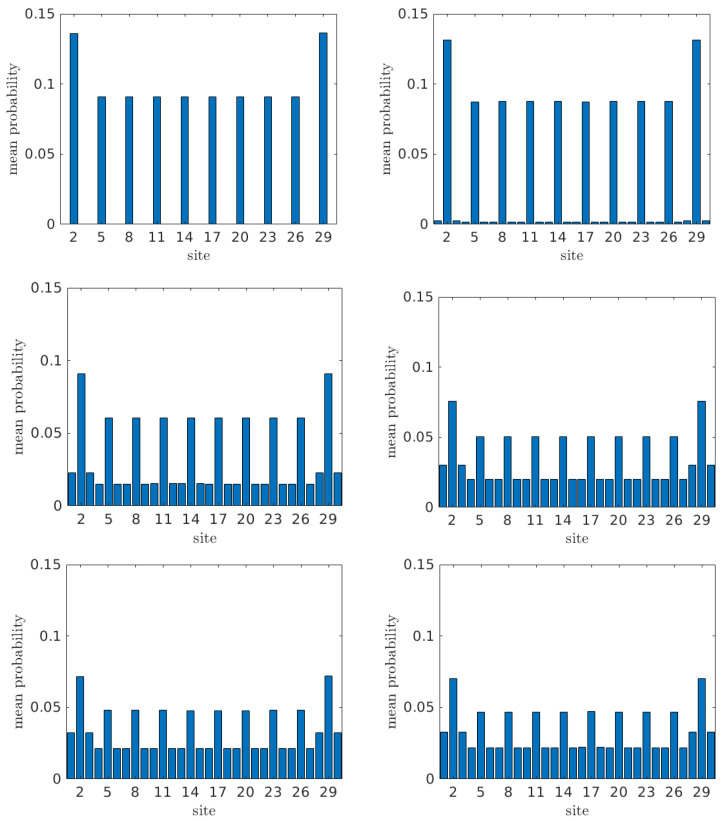
The mean-over-time probability at each site β, $\langle |C_{\beta }\left(t\right){|}^{2}\rangle$ for a G... homopolymer with N=10 for $t_{S}=0.0,0.1,0.5,1.0,1.5,2.0$ eV (from left top to bottom right) with initial carrier placement at the base pair of the first monomer (α = 2).

**Figure 5:**
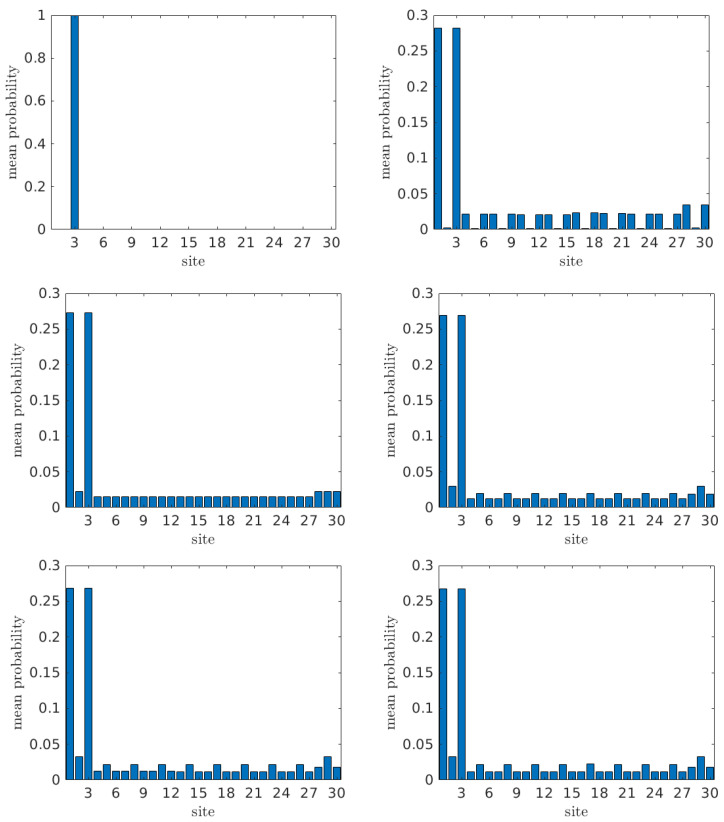
The mean-over-time probability at each site β, $\langle |C_{\beta }\left(t\right){|}^{2}\rangle$ for a G... homopolymer with N=10 for $t_{S}=$ 0.0, 0.1, 0.5, 1.0, 1.5, and 2.0 eV (from left top to bottom right) with initial hole placement at the “right” deoxyribose of the first monomer (α=3).

In Figure 4, we illustrate $\langle |C_{\beta }\left(t\right){|}^{2}\rangle$ for all sites for a G... homopolymer with N=10, and for $t_{S}=$ 0.0, 0.1, 0.5, 1.0, 1.5, and 2.0 eV, with initial hole placement at the base pair of the first monomer (i.e., α=2). Similarly, in Figure 5 we present the same quantities but for initial hole placement at the “right” deoxyribose of the first monomer (i.e., α=3).

As evident from Figure 4, when the carrier is initially placed at the first base pair (α=2), it can readily be transferred to other base pairs. For $t_{S}=0$, the values of $\langle |C_{\beta }\left(t\right){|}^{2}\rangle$ for base pairs are the ones expected from the wire model [20], with a characteristic *palindromicity*, while all deoxyribose sites remain vacant. Introducing $t_{S}\neq 0$, we observe transfer to deoxyriboses while palindromicity still holds. Transfer to deoxyriboses is weaker than to base pairs, and increases with $t_{S}$. Furthermore, it is remarkable that the concept of “favored” and “rest” monomers of the wire model [20] holds here as well with a slight modification: within the wire model [20], the monomer is only the base pair; on the contrary, within the FWM, the carrier can move between the three sites of the monomer, i.e., the base pair and the two deoxyriboses at either side of the base pair.

For homopolymers, for initial carrier placement at a particular monomer, we obtain $\frac{1}{2(N+1)}$ additional mean-over-time probabilities at the monomer where the initial placement is made *and also* at its symmetric monomer relative to the polymer center. For *N* odd, and for initial carrier placement at the central monomer, that central monomer receives $\frac{2}{2(N+1)}$ additional mean-over-time probabilities. We denote the mean-over-time probabilities at the “favored” and at the “rest” monomers by ψ and χ, respectively, and then $\psi =\chi +\frac{1}{2(N+1)}$ (or $\psi =\chi +\frac{2}{2(N+1)}$ for *N* odd and initial placement at the central monomer). Since the sum of all the mean-over-time probabilities is 1, we obtain

(39) $$\psi =\frac{3}{2(N+1)},\;\chi =\frac{1}{N+1}.$$

(or $\psi =\frac{2}{N+1}$, $\chi =\frac{1}{N+1}$ for *N* odd and initial carrier placement at the central monomer).

In the example of Figure 4, where N=10, for any choice of $t_{S}$, we obtain $\sum_{1}^{3}\langle |C_{\beta }\left(t\right){|}^{2}\rangle =3/22=0.13\bar{63}$ for the initial monomer (where the carrier is initially placed), $\sum_{4}^{27}\langle |C_{\beta }\left(t\right){|}^{2}\rangle =\left(1/11\right)\times \left(10-2\right)=0.\bar{72}$ for the middle monomers (channel), and $\sum_{28}^{30}\langle |C_{\beta }\left(t\right){|}^{2}\rangle =3/22=0.13\bar{63}$ for the last monomer (where we want the carrier to arrive).

Comparing between Figure 4 and Figure 5, we observe that placing the carrier initially at the base pair of the first monomer (α=2, Figure 4) leads to much more efficient transfer to other monomers along the polymer than does placing it initially at one of the deoxyriboses of the first monomer (α=3, Figure 5; the results are identical for α=1). For initial placement at a deoxyribose of the first monomer, when $t_{S}=0$, there is no transfer at all. Introducing interaction between base pairs and deoxyriboses ($t_{S}\neq 0$), we observe that, for small $t_{S}$, the carrier is transferred with greater probability to deoxyriboses than to base pairs. The first two deoxyriboses are clearly favored, while the last two deoxyriboses have slightly larger mean-over-time probabilities than the ones of the channel monomers, while a small amount goes to the base pairs. Increasing $t_{S}$ to 0.5 eV, the deoxyriboses of the first monomer (ν=1) still remain favored, whereas, for sites beyond ν=1, the probabilities are similar for both deoxyriboses and base pairs. When further increasing $t_{S}$ to 1 eV and beyond, while the deoxyriboses of ν=1 still remain favored, the base pairs of ν≠1 display larger probabilities than the corresponding deoxyriboses. In general, for $t_{S}\neq 0$, careful examination reveals that the first monomer is emphatically favored, while the last monomer receives a somehow larger probability than the middle ones. In specific, for $t_{S}\neq 0$,

(40) $$\omega =\frac{2N+5}{4(N+1)},\;\chi =\frac{1}{2(N+1)},\;\phi =\frac{3}{4(N+1)}$$

for the first, a middle, and the last monomer, respectively. In the example of Figure 5, where N=10, for any choice of $t_{S}$, we obtain $\sum_{1}^{3}\langle |C_{\beta }\left(t\right){|}^{2}\rangle =25/44=\frac{2N+5}{4(N+1)}$ for the initial monomer (where the carrier is initially placed). $\sum_{1}^{3}\langle |C_{\beta }\left(t\right){|}^{2}\rangle =25/44=\frac{2N+5}{4(N+1)}$ for the middle monomers (channel), and $\sum_{28}^{30}\langle |C_{\beta }\left(t\right){|}^{2}\rangle =3/44=\frac{3}{4(N+1)}$ for the last monomer (where we want the carrier to arrive).

As a final remark, comparing Equations (39) and (40), we conclude that, for homopolymers with a given *N*, the probabilities to find the hole at the first, a middle, and the last monomer for initial hole placement at the base pair of the first monomer were $\frac{6}{2N+5}$ times, two times, and two times, respectively, the one for initial hole placement at one of the deoxyriboses of the first monomer. The size-dependence of the aforementioned ratio for the first monomer indicates that, for large polymers, it becomes increasingly difficult for a hole to be transferred from the first monomer if it was initially placed at one of its deoxyriboses.

#### 3.1.4. Frequency Content

As displayed in Figure 1, we are studying finite systems spanning the *x*- and *y*- axes, inside which the carrier probability oscillates between various sites. The frequency content of these carrier probability oscillations characterizes the carrier movement. The total dipole moment oscillates as well; hence, it can also be used to characterize carrier movement.

Higher frequencies mean faster carrier movement within the system. The frequency content can be detected via either analytical [cf., Equation (33)] or numerical (Fast Fourier Transform, FFT) Fourier transform of the time-dependent probabilities and of the dipole moment oscillations. Details can be found in Ref. [34]. The overall picture from charge oscillations and from dipole moment oscillations is that there are: (a) lower-frequency (crudely below 100 THz) oscillations along the polymer (*x*-axis), i.e., due to the sequence of monomers; and (b) higher-frequency (crudely 700–800 THz) oscillations across the polymer (*y*-axis), i.e., within monomers.

**Figure 6:**
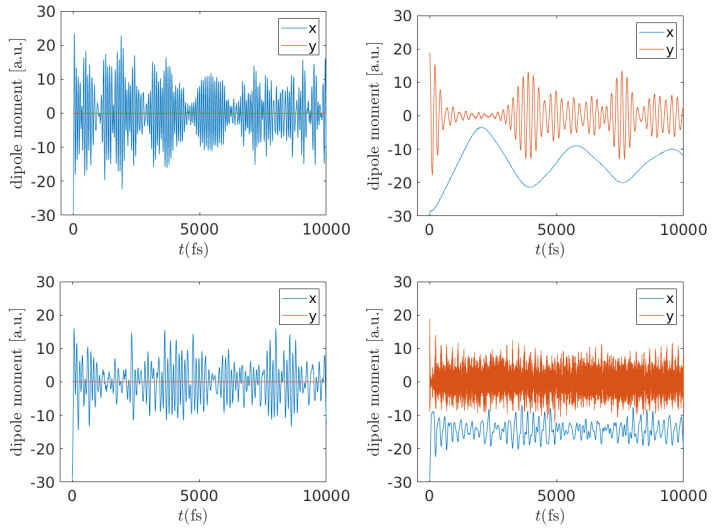
Dipole moment oscillations (in atomic units) for a G... homopolymer N=10. Initial carrier placement at site α=2 (**left**) and site α=3 (**right**). Top row: $t_{S}=0.1$ eV. Bottom row: $t_{S}=1$ eV.

The character of these oscillations is better revealed in Figure 6, where the dipole moment components are presented as functions of time. The *x*- and *y*- axes are defined so that x=0 coincides with the center of the molecule along its length and so that y=0 coincides with the center of the molecule across its width (i.e., so that the *y*-center is always at σ=2), according to Figure 1. For α=2 (left), the dipole moment oscillates solely along the macromolecule (*x*-axis); due to the symmetry of our FWM, no dipole moment occurs across the molecule, cf. also Figure 4. For α=3 (right), carrier movement occurs mainly across the macromolecule (*y*-axis); there is also some activity along the macromolecule, but the carrier basically remains at the negative *x*- region, i.e., mainly at ν=1 (negative *x* corresponds to the first half of the homopolymer, cf. Figure 1), cf. also Figure 5.

**Figure 7:**
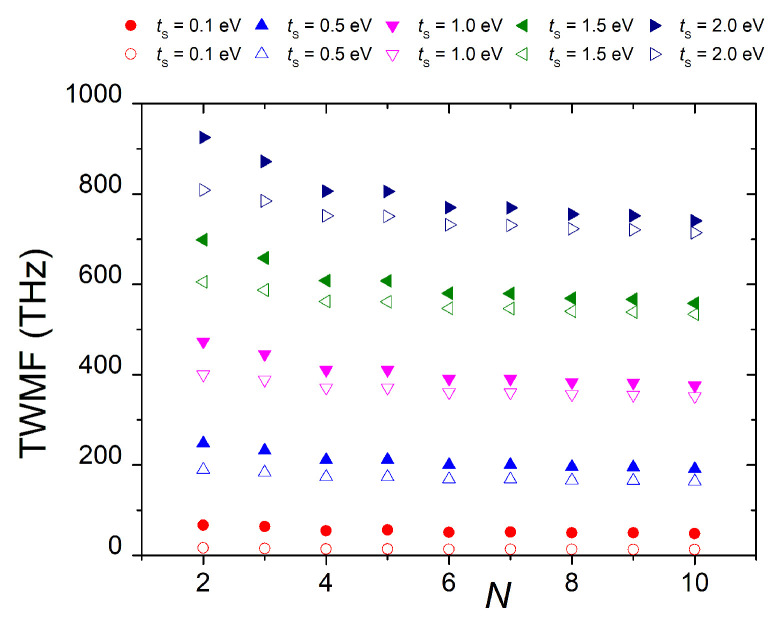
TWMF of G... homopolymers for α=2 (filled symbols) and α=3 (open symbols) for various $t_{S}$ values.

In Figure 7, the TWMF is depicted as a function of *N* for five values of $t_{S}$ and for segments with N=2,…,10. The carrier is, again, initially placed either at site α=2 (filled symbols) or at site α=3 (empty symbols). Red, blue, pink, green, and dark blue symbols correspond to $t_{S}=0.1,0.5,1,1.5,$ and 2 eV, respectively. Increasing $t_{S}$, i.e., when strengthening the connection between base pairs and deoxyriboses, the TWMF increases. Additionally, as expected, we observe a decrease in the TWMF as *N* increases, i.e., as the fishbone chord becomes longer. Moreover, when increasing $t_{S}$, this decrease in the TWMF becomes steeper. Finally, comparing the TWMFs for the two different initial conditions, i.e., α=2 (filled symbols) vs. α=3 (empty symbols), we observe that, for given *N* and $t_{S}$, the TWMF is larger for α=2, i.e., the overall transfer is faster for initial placement at the base pair of the first monomer than for initial placement at one of its deoxyriboses.

#### 3.1.5. Mean Transfer Rates

**Figure 8:**
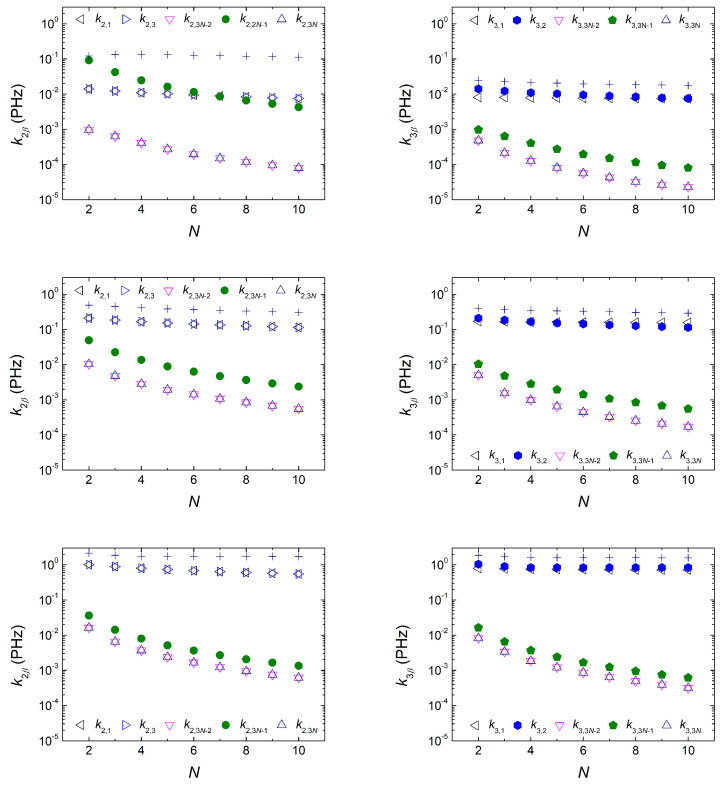
The mean transfer rates, $k_{\alpha \beta }$ ($k_{\alpha ,\beta }$), from site α to sites β= 1, 2 (or 3), 3N−2, 3N−1, and 3N, increasing the number of monomers in the polymer, *N*, for a G.. homopolymer. **Left**: α=2. **Right**: α=3. First, second, and third row corresponds to $t_{S}=0.1$, 0.5, and 2 eV, respectively. $\sum_{\beta \neq \alpha }^{3N}k_{\alpha \beta }$ is also shown (+ symbols).

In Figure 8, we illustrate the hole mean transfer rate $k_{\alpha \beta }$ as a function of *N* for initial carrier placement at sites α=2 (base-pair of the first monomer, left column) and α=3 (“right” deoxyribose of the first monomer, right column) for homopolymers with N=2,..., 10. We focus on the rates from sites of the first monomer (ν=1) to sites of the last monomer (ν=N) for varying $t_{S}=$ 0.1, 0.5, and 2 eV. As expected, $k_{\alpha \beta }$ decreases with *N*. The decrease in *k* to sites of the last monomer is more acute.

On the left panels (initial oxidation at the first base pair, α=2), increasing $t_{S}$, the rates involving the first monomer, $k_{2,1}$ and $k_{2,3}$, increase noticeably. Similarly, although less pronounced, the rates involving the deoxyriboses of the last monomer, $k_{2,3N-2}$ and $k_{2,3N}$, increase. These effects lead to a decrease in the transfer rate between the edge base pairs, $k_{2,3N-1}$. On the right panels (initial oxidation at the “right” deoxyribose of the first monomer, α=3), increasing $t_{S}$, the rates involving the first monomer, $k_{3,1}$ and $k_{3,2}$, increase noticeably.

Similarly, although less pronounced, all rates involving the last monomer, i.e., $k_{3,3N-2}$, $k_{3,3N-1}$$k_{3,3N}$ increase, as well. Comparing the left and right panels of Figure 8, it can be observed that, for given *N* and $t_{S}$, the coherent mean transfer rates involving end-to-end transfer are larger for initial hole placement at the base pair of the first monomer compared to initial hole placement at a deoxyribose of the first monomer, which is in accordance with the discussion of Section 3.1.3. In Figure 8, the sum of all $k_{\alpha \beta }$, i.e., $\sum_{\beta \neq \alpha }^{3N}k_{\alpha \beta }$ is also displayed. In the first row, $|t_{S}\left|=\right|t_{\mathrm{GG}}|$; in the second row, $|t_{S}\left|=5\right|t_{\mathrm{GG}}|$; and in the third row (an exaggerated case), $|t_{S}\left|=20\right|t_{\mathrm{GG}}|$. An increasing trend with $t_{S}$ is observed in this quantity, as well.

**Figure 9:**
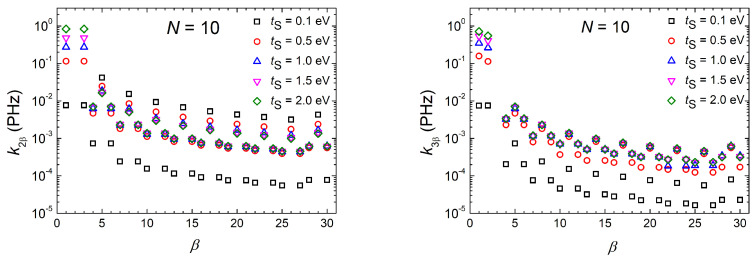
Variation of the mean transfer rates, $k_{\alpha \beta }$, from initial site α=2 or 3 to sites β=1,…,30 (β≠α) for a G... homopolymer made of N=10 monomers for five values of $t_{S}$. **Left**: α=2. **Right**: α=3.

In Figure 9, we present the variation of the mean transfer rate, $k_{\alpha \beta }$, from initial sites α=2 and α=3 to sites β=1,…,30 (β≠α) for a homopolymer made of N=10 monomers for five different values of $t_{S}$. The transfer rates to the sites of the same monomer form triplets (e.g., $\left\{k_{24},k_{25},k_{26}\right\}$). Increasing $t_{S}$, the values of the same triplet converge. For α=3, the *k* values of the triplet of each monomer are closer than for α=2.

In experiments, it is common to fit transfer rates using $k=k_{0}\exp\left(-\beta r\right)$, where *r* is the traveled distance, or $k=k_{0}^{\prime }{\left(N-1\right)}^{-\eta }$, where N−1 is the number of steps (i.e., for a polymer of *N* monomers, the number of steps is N−1). We have performed similar fits in the past [18,20,21]. Homopolymers (G... or A...) have the same β or η, but $k_{0}$ or $k_{0}^{\prime }$ differ (they depend on the specific homopolymer and on whether we deal with HOMO or LUMO). In these works, for homopolymers, we find β≈0.2 Å${}^{-1}$ and η≈1.3 (if we fit as $k=k_{0}^{\prime }{\left(N-1\right)}^{-\eta }$) or $\eta^{\prime }\approx 2$ (if we fit as $k=k_{0}^{\prime }N^{-\eta^{\prime }}$).

In the experiments of Ref. [35], the authors found results for homooligomers, where β≈0.2 Å${}^{-1}$ and η≈1.5, i.e., our above-mentioned works predicted similar β and η. The β values of the transfer rates $k_{2,3N-1}$ for the FWM, i.e., for initial hole placement at the first base pair and receipt of the hole at the last base pair, for $t_{S}=$ 0.1, 0.5, and 2 eV, respectively, are 0.178 ± 0.015, 0.175 ± 0.016, and 0.218 ± 0.021, and the η values are 1.248 ± 0.036, 1.238 ± 0.033, and 1.402 ± 0.019, respectively, (the $\eta^{\prime }$ values are 1.895 ± 0.004, 1.879 ± 0.013, and 2.164 ± 0.037, respectively). Hence, although β and η values are affected by $t_{S}$, it is not safe to draw conclusions about the value of the latter based on transfer rate fits.

### 3.2. Dimers

**Figure A1:**
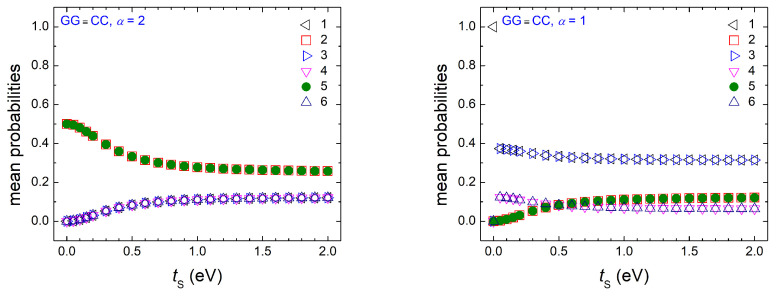

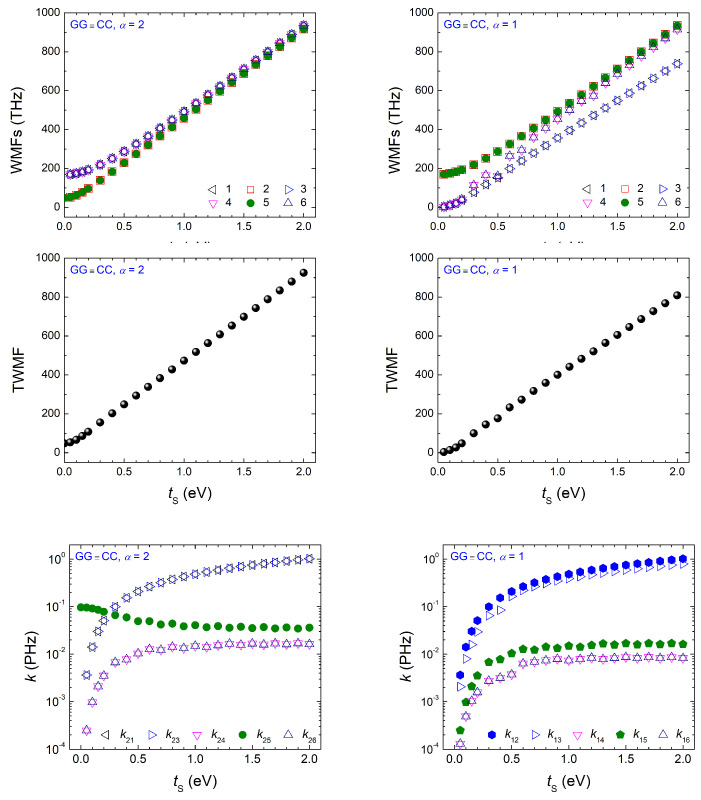
A dimer made of identical monomers and purine on purine: GG≡CC. Initial oxidation occurs either at the first base pair (α=2, **left column**) or at the first deoxyribose (α=1, **right column**). The mean-over-time probabilities to find the hole at each dimer site (first row), WMFs of all sites (second row), TWMF (third row), and transfer rates from site to site are presented as functions of $t_{S}$.

**Figure A2:**
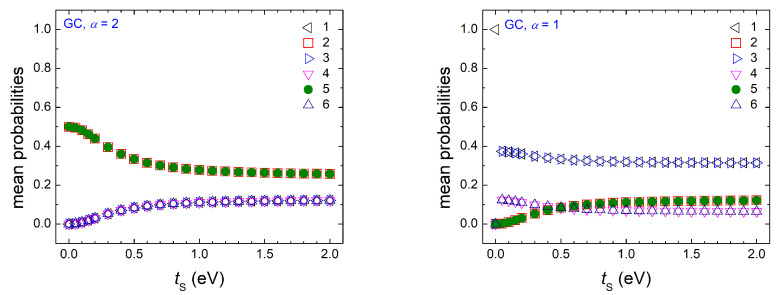

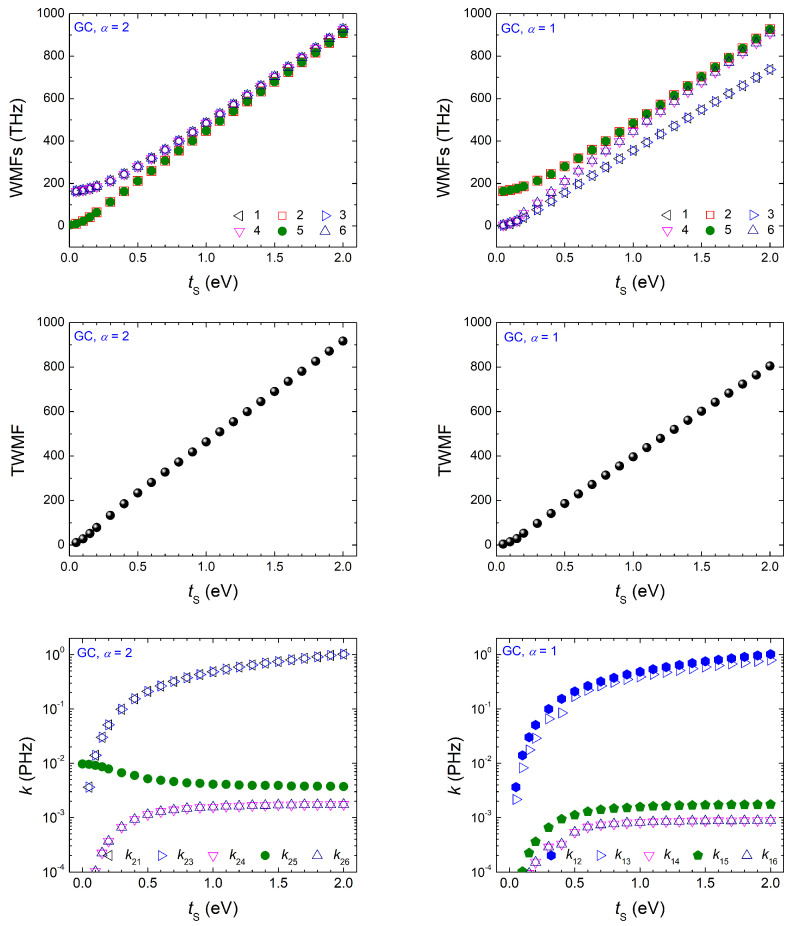
A dimer made of identical monomers and purine on pyrimidine: GC dimer. Initial oxidation occurs either at the first base pair (α=2, **left column**) or at the first deoxyribose (α=1, **right column**). The mean-over-time probabilities to find the hole at each dimer site (first row), WMFs of all sites (second row), TWMF (third row), and transfer rates from site to site are presented as functions of $t_{S}$.

**Figure A3:**
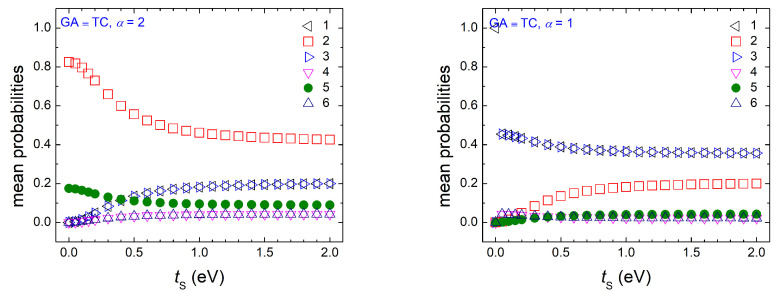

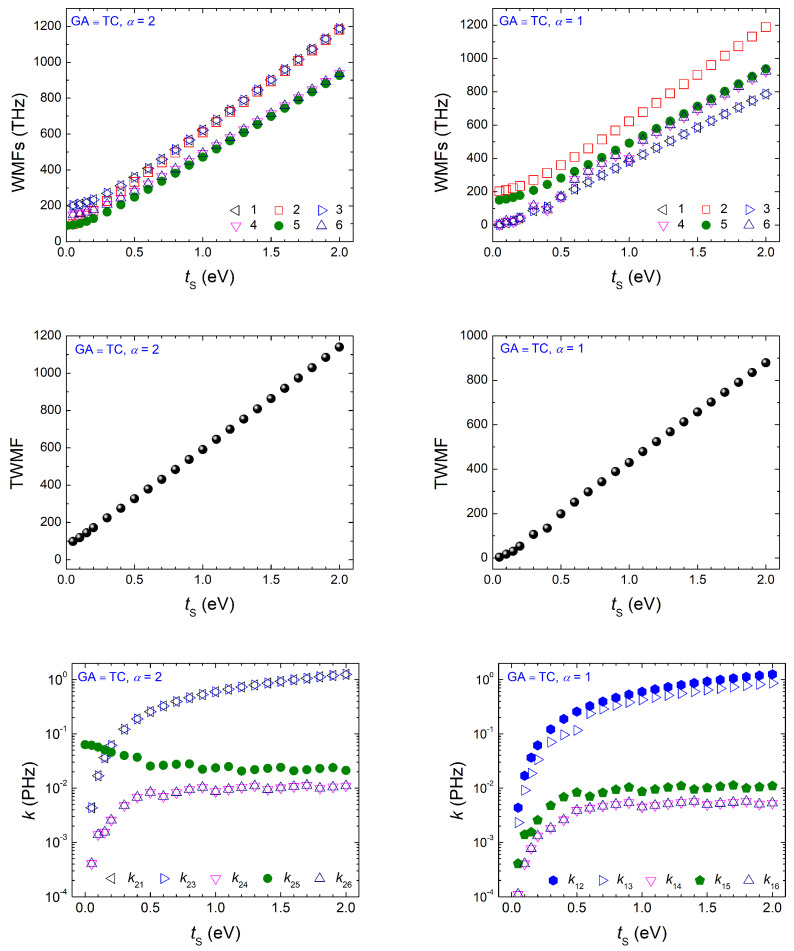
A dimer made of different monomers: GA ≡ TC dimer. Initial oxidation occurs either at the first base pair (α=2, **left column**) or at the first deoxyribose (α=1, **right column**). The mean-over-time probabilities to find the hole at each dimer site (first row), WMFs of all sites (second row), TWMF (third row), and transfer rates from site to site are presented as functions of $t_{S}$.

In Appendix A, hole-transfer-related quantities are presented as functions of $t_{S}$ for one characteristic case of each dimer type (i.e., made of the same monomer with purine on purine, made of the same monomer with purine on pyrimidine, and made of different monomers) for initial oxidation at the first base pair (left columns of Figure A1, Figure A2 and Figure A3) and at one deoxyribose of the first monomer (right columns of Figure A1, Figure A2 and Figure A3).

For all dimer types, for initial oxidation at the first base pair (α=2) and for $t_{S}=0$ (no interaction between base pairs and deoxyriboses), the mean-over-time probabilities and coherent mean transfer rates are identical to those predicted by the simple wire model [18]. As $t_{S}$ increases, for initial oxidation of the first base pair (deoxyribose), the mean-over-time probabilities to find the hole at a deoxyribose (base pair) are increased (decreased). As $t_{S}$ becomes larger, all values converge. Overall, the amount of charge that is transferred to the second monomer is large when the dimer is made up of the same monomer. Additionally, in accordance with the above discussion for homopolymers (cf., Section 3.1.3), initial oxidation on a base pair is more efficient than initial oxidation on a deoxyribose.

Regarding the frequency content of hole transfer, increasing $t_{S}$ leads to an increase in all WMFs and of the TWMF in all cases; after a threshold value of ≈0.2 eV, this increase is linear. Furthermore, in all cases displayed, for the initial oxidation of the first base pair (deoxyribose), the WMFs of the deoxyriboses (base pairs) are larger than the those of the base pairs (deoxyriboses). Additionally, in all cases displayed, the TWMF is larger for the initial oxidation of the base pairs than it is for the initial oxidation of a deoxyribose. Finally, as far as transfer rates are concerned, similar to homopolymers (cf., Section 3.1.5), for initial oxidation at the first base pair, increasing $t_{S}$ increases the rates involving the first monomer and the rates involving the deoxyriboses of the second monomer, leading to a decrease in the rate involving the base pairs; for small values of $t_{S}$, inter-base-pair transfer rates are dominant.

On the contrary, for initial placement at a deoxyribose, all rates increase with $t_{S}$. As in homopolymers, for a given $t_{S}$, the coherent mean transfer rates involving end-to-end transfer are larger for initial hole placement at the base pair of the first monomer compared to initial hole placement at a deoxyribose of the first monomer.

## 4. Conclusions

We studied the electronic structure and the hole-transfer properties of DNA homopolymers and of all types of dimers within the fishbone-wire variant of the TB method, using known HOMO on-site energies for the base pairs and deoxyriboses as well as interaction integrals between successive base pairs. We treated the interaction integral between base pairs and deoxyriboses ($t_{S}$) as a variable and examined the influence of its value on the results for both the time-independent and time-dependent problems in the absence of backbone disorder.

Studying the time-independent problem for homopolymers, we compared the eigenenergies for different sequence lengths and for different values of $t_{S}$. Switching on the interaction between base pairs and deoxyriboses, we moved from the picture obtained by the wire model for base pairs [20] plus isolated deoxyriboses to the formation of three subbands: the middle one is made of *N* degenerate eigenenergies (at $E_{S}$); the other two are composed of *N* eigenenergies each, above and below $E_{S}$, with no symmetry relative to some center. We also obtained the DOS for large *N* to clarify their form. As $t_{S}$ increases, the “wire” subband (at higher energies) is displaced due the interactions between base pairs and deoxyriboses. The two non-degenerate subbands move further away from each other, and their width gradually changes.

We also studied the time-dependent problem for homopolymers and discussed quantities related to hole transfer. Examining the mean-over-time probability to find the hole at each site for the initial placement at the first base pair, we showed that it can readily be transferred to the remaining base pairs. Edge monomers are clearly preferred, and the probabilities display palindromicity.

When increasing the interaction between base pairs and deoxyriboses, increased transfer to deoxyriboses occurs. On the contrary, for initial placement at one of the deoxyriboses of the first monomer, the deoxyriboses of the first monomer are clearly preferred. When increasing $t_{S}$, the transfer to base pairs increases. However, the value of $t_{S}\neq 0$ determines only the probability distribution *within* each monomer, not *between* monomers; the latter is solely dependent on *N*. We also showed that, for given *N* and $t_{S}$, initial placement at a base pair doubles all the probabilities to find the hole elsewhere in the segment compared to initial placement at a deoxyribose; it becomes increasingly difficult with *N* for a hole to be transferred from the first monomer, had it been initially placed at one of its deoxyriboses.

Additionally, dipole moment components can clearly show the type of carrier movement: for initial placement at the first base pair, the dipole moment oscillates solely along the macromolecule, while, for initial placement at a deoxyribose site, it mainly oscillates across the macromolecule. We also examined the frequency content of carrier movement, using analytical and numerical Fourier transforms and obtained the WMF of all sites and the TWMF of the polymer. These quantities can also be used to characterize the readiness of coherent charge transfer within the polymer. The TWMF decreases with polymer size, increases with $t_{S}$, and is larger for initial placement at a base pair compared to initial placement at a deoxyriboze site.

The mean transfer rate between a site of the first monomer and a site of the last monomer decreases with *N*, as expected. The increase in $t_{S}$ increases the sum of all mean transfer rates, i.e., $\sum_{\beta \neq \alpha }^{3N}k_{\alpha \beta }$. For given $t_{S}$ and *N*, the coherent mean transfer rates involving end-to-end transfer are larger for initial hole placement at the base pair of the first monomer compared to initial hole placement at a deoxyribose of the first monomer, in accordance with what happens with the mean probabilities. Similar conclusions can be reached when studying hole transfer in all categories of DNA dimers, i.e., made up of identical monomers with purine on purine, made up of identical monomers with purine on pyrimidine, and made up of different monomers.

## Figures and Tables

**Table 1:** Interaction integrals for hole transfer between consecutive base pairs; values taken from Ref. [18]. All values are given in meV. The notation XY means that dimers are named using their bases in the 5${}^{\prime }$–3${}^{\prime }$ direction. Hence, e.g., TG≡CA, since the sequence TG in the 5${}^{\prime }$–3${}^{\prime }$ direction of one strand gives the same dimer with the sequence CA in the 5${}^{\prime }$–3${}^{\prime }$ direction of the complementary strand.

Dimer	*t*	Dimer	*t*	Dimer	*t*	Dimer	*t*
AA≡TT	20	GG≡CC	100				
AT	−35	GC	−10	AG≡CT	30	TC≡GA	110
TA	−50	CG	50	AC≡GT	−10	TG≡CA	10

## Data Availability

Data available upon reasonable request.

## Abbreviations

The following abbreviations are used in this manuscript:

TB	Tight Binding
FWM	Fishbone-Wire Model
DOS	Density of States
HOMO	Highest Occupied Molecular Orbital
LUMO	Lowest Unoccupied Molecular Orbital
WMF	Weighted Mean Frequency
TWMF	Total Weighted Mean Frequency

## Appendix A. Results for All Dimer Types

Additional results and details can be found in Ref. [36].
